# Modeling and Simulation of the Hysteretic Behavior of Concrete under Cyclic Tension–Compression Using the Smeared Crack Approach

**DOI:** 10.3390/ma16124442

**Published:** 2023-06-17

**Authors:** Pei Zhang, Shenshen Wang, Luying He

**Affiliations:** 1 College of Mechanical Engineering and Mechanics, Xiangtan University, Xiangtan 411105, China; 2 College of Civil Engineering, Xiangtan University, Xiangtan 411105, China; 202121572230@smail.xtu.edu.cn (S.W.); 202121572183@smail.xtu.edu.cn (L.H.)

**Keywords:** concrete, hysteretic behavior, energy dissipation, cyclic loading, smeared crack model, numerical simulation, dynamic behavior

## Abstract

Concrete structures under wind and earthquake loads will experience tensile and compressive stress reversals. It is very important to accurately reproduce the hysteretic behavior and energy dissipation of concrete materials under cyclic tension–compression for the safety evaluation of concrete structures. A hysteretic model for concrete under cyclic tension–compression is proposed in the framework of smeared crack theory. Based on the crack surface opening–closing mechanism, the relationship between crack surface stress and cracking strain is constructed in a local coordinate system. Linear loading–unloading paths are used and the partial unloading–reloading condition is considered. The hysteretic curves in the model are controlled by two parameters: the initial closing stress and the complete closing stress, which can be determined by the test results. Comparison with several experimental results shows that the model is capable of simulating the cracking process and hysteretic behavior of concrete. In addition, the model is proven to be able to reproduce the damage evolution, energy dissipation, and stiffness recovery caused by crack closure during the cyclic tension–compression. The proposed model can be applied to the nonlinear analysis of real concrete structures under complex cyclic loads.

## 1. Introduction

The safety assessment of concrete structures subjected to cyclic loading such as seismic excitation requires realistic constitutive models to reproduce the real behavior of the materials. Due to the low tensile strength, the concrete subjected to seismic load usually presents softening behavior in tension and hysteretic behavior in tension–compression reversals. As a result, the hysteretic model for concrete plays a significant role in determining the seismic responses of concrete structures including the deformation and energy evolution. However, due to the lack of experimental data, studies on modeling the hysteresis behavior of concrete under cyclic tension and tension–compression reversal are scarce compared to those on cyclic compression [1,2,3,4,5].

At present, most concrete constitutive models considering the cyclic tension assumed that the unloading path coincides with the reloading path, neglecting the hysteretic behavior and energy dissipation caused by the crack opening and closing [6,7,8,9,10]. Several researchers [11,12,13,14] have suggested various modeling strategies to replicate the hysteretic stress–strain curves of concrete under cyclic loading, including the complete unloading–reloading path and the partial unloading–reloading path.

Yankelevsky and Reinhardt [11] proposed a focal model to reproduce the hysteretic curves through a series of focal points. These predefined focal points govern the unloading and reloading paths either by the rays from themselves or by their stress level. When all the focal points are determined, the complete unloading–reloading curves can be plotted through a simple graphical process. Although the focal model performed well in replicating the test results, it is thought that the concept of a focal point, which is solely derived from the graphic feature, lacks physical significance and that the process for determining unloading and reloading paths is excessively complex.

Chang and Mander [12] proposed a rule-based model to simulate the hysteretic behavior of confined and unconfined concrete in both cyclic compression and tension. Fifteen unloading and reloading paths governed by fifteen different rules are prescribed in the model. The unloading and reloading curves in the model are characterized by the polynomial equation. The rule-based model is capable to simulate the hysteretic curve of concrete under various loading conditions. However, due to the overabundance of rules and paths in the model, the parameters of the unloading and reloading paths are difficult to calibrate, and the calculation is very time-consuming.

Aslani and Jowkarmeimandi [13] proposed a constitutive model for concrete under cyclic loading based on the findings of previous experimental and analytical studies. The model for concrete subjected to monotonic and cyclic loading comprises four components: an envelope curve, an unloading curve, a reloading curve, and a transition curve. A crack-closing model is suggested, in which the crack-closure mechanism is governed by the crack-closure stress. The unloading curve in compression is described by a power equation, whereas the reloading curve is described by a linear equation. However, the model fails to account for hysteretic behavior and energy dissipation during cyclic tension due to the use of the same path for both unloading and reloading processes in tension.

The most popular model for modeling the mechanical behavior of concrete is the plastic damage model, which can replicate concrete’s irreversible deformation and stiffness degradation under monotonic and cyclic loads with confinement [8,9,10]. The unloading–reloading law in the plastic damage model is usually controlled by the damage modulus and the residual plastic strain. Chen et al. [14] proposed analytical expressions introducing damage index to describe the response of concrete to cyclic loadings. A power type function is selected to model the unloading and reloading curve and the power is expressed as a function of the damage index and the strain rate. McCall and Guyer [15] suggested that cracks inside nonlinear mesoscopic elastic material such as sandstone were denoted as hysteretic mesoscopic elastic units. Based on the assumption, Preisach–Mayergoyz (P-M) model was used to simulate the phenomenon of the hysteretic loop in concrete under cyclic tension and alternating tensile–compressive loading [16,17]. However, the P-M model can only reproduce the hysteretic curves and cannot simulate the process of concrete crack propagation under cyclic tension.

Based on the continuum damage theory, Long et al. [18,19] developed an improved anisotropic damage model to simulate the nonlinear behavior of concrete by proposing the independent tensile and compressive damage evolution laws. The hysteretic behavior is described by a nonlinear unloading path and a linear reloading path. A stiffness recovery coefficient is defined to model the stiffness recovery phenomenon caused by crack-closing. However, the simulated results of concrete hysteretic behavior under tension–compression reversals are not satisfactory.

Liu et al. [20] combined the loading and unloading characteristic points and the loading and unloading paths in the hysteretic rules to construct a four-parameter plastic damage model considering the hysteretic effect under cyclic loading. The reloading curve is simplified to linear and the unloading curve is represented by an exponential equation reported in the literature [5]. Nonlinear characteristics of concrete such as stiffness degradation, strength softening, irreversible plastic deformation, and hysteresis effects under cyclic loading were simulated by the model. However, the model assumes that damage accumulates during the unloading process and remains unaltered during the reloading process, which is inconsistent with the concrete damage mechanism.

In this paper, an efficient model capable of predicting the hysteretic behavior of concrete under cyclic tension and tension–compression reversals is proposed within the framework of smeared crack theory. By decomposing the total strain into the elastic strain and crack strain, it is possible to directly model the crack surface behavior that causes the hysteretic phenomenon. Straight lines are adopted to describe the unloading and reloading paths, and several necessary hysteretic rules are proposed based on the closing–opening mechanism of the crack surface. Furthermore, the crack surface-closure effect during the tension–compression reversals is considered by the definition of crack-closure stress parameters. Compared to the existing hysteretic models, only two model parameters are needed in the proposed model to simulate the complete and partial unloading–reloading curves. The model has been validated by comparison with available test results under different loading conditions.

## 2. Theoretical Framework of Smeared Crack Model

### 2.1. Overview of the Smeared Crack Model

The nonlinear behavior of concrete is mainly caused by the initiation and propagation of cracks. In the finite element simulation, the description of concrete cracks generally has two ways: one is the discrete crack method [21,22,23,24] and the second is the smeared crack method [25,26,27,28,29]. The discrete crack model assumes that cracks appear on the element boundaries. Once the crack generates, new nodes are added and the mesh is re-divided to produce new element boundaries. In this way, the geometric discontinuity caused by the crack is directly characterized, and the position, shape, and width of the crack can be clearly described.

**Figure 1:**
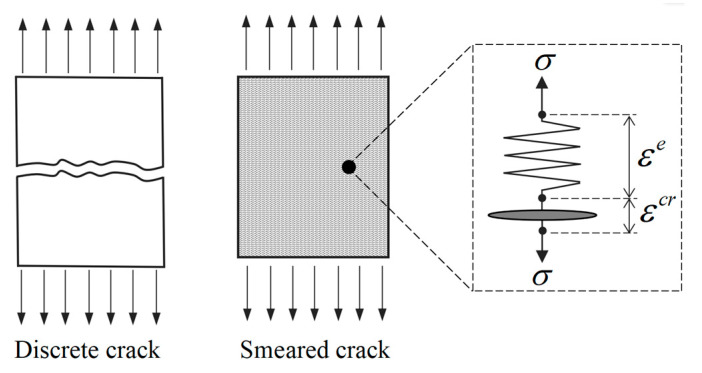
A schematic diagram of the discrete crack and smeared crack.

The smeared crack method assumes that local discontinuities caused by cracks are uniformly distributed in the fracture zone of the finite element (Figure 1). Based on this assumption, the relative displacement between the crack surfaces can be characterized by the crack strain, and the constitutive behavior of the cracked concrete can be directly modeled in terms of the stress–strain relations. Contrary to the discrete crack concept, the smeared crack concept fits the nature of the finite element displacement method, as the continuity of the displacement field remains intact [30]. The main ingredients of the smeared crack model are introduced here, and a full description can be found in the literature [29].

**Figure 2:**
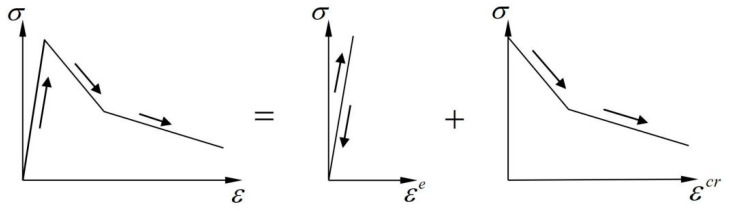
Decomposition of the stress–strain relationship for cracked concrete.

The smeared crack model decomposes the total strain in the fracture zone into two parts: elastic strain and crack strain. The elastic strain corresponds to the deformation yielded by the uncracked portion, and the crack strain corresponds to the equivalent deformation generated by the crack opening. In the one-dimensional case, the schematic diagram of strain decomposition is illustrated in Figure 1, and the corresponding formula can be written as:

(1) $$\varepsilon =\varepsilon^{e}+\varepsilon^{cr}$$

where $\varepsilon^{e}$ represents the elastic strain and $\varepsilon^{cr}$ represents the crack strain. The major advantage of this decomposition is that it allows the crack surface behavior to be treated separately from the constitutive behavior of the uncracked concrete [30]. As a result, the stress–strain relationship of the fracture zone in concrete can be treated as the superposition of the elastic part and the cracked part, as illustrated in Figure 2.

### 2.2. Constitutive Relations in Local Coordinate System

In the multiaxial stress state, the strain decomposition can be written in a tensor form as:

(2) $$\varepsilon =\varepsilon^{e}+\varepsilon^{cr}$$

The corresponding incremental form is:

(3) $$\Delta \varepsilon =\Delta \varepsilon^{e}+\Delta \varepsilon^{cr}$$

**Figure 3:**
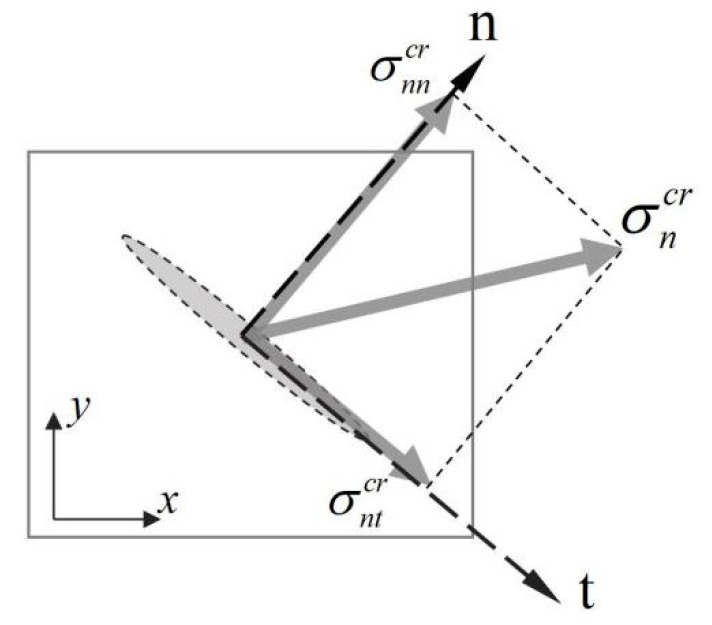
Local coordinates system normal to the crack surface.

When the first principal stress exceeds the tensile strength, the crack generates and the crack surface is perpendicular to the direction of the first principal stress. In the two-dimensional case, the behavior of the crack surface can be clearly described in the local coordinate system as illustrated in Figure 3, where the subscript *n* represents the direction normal to the crack surface, and the subscript *t* represents the direction tangential to the crack surface. Projecting the stress on the crack surface $\sigma_{n}^{cr}$ onto the local coordinate system, the corresponding normal stress component $\sigma_{nn}^{cr}$ and tangential stress component $\sigma_{nt}^{cr}$ can be obtained. Correspondingly, the cracking strain on the crack surface $\varepsilon_{n}^{cr}$ can be decomposed into the normal cracking strain $\varepsilon_{nn}^{cr}$ and the tangential cracking strain $\gamma_{nn}^{cr}$ in the local coordinate system. In the smeared crack model, the normal cracking strain $\varepsilon_{nn}^{cr}$ corresponds to the open displacement of the crack surface, and the tangential cracking strain $\gamma_{nn}^{cr}$ corresponds to relative slip displacement on the crack surface.

**Figure 4:**
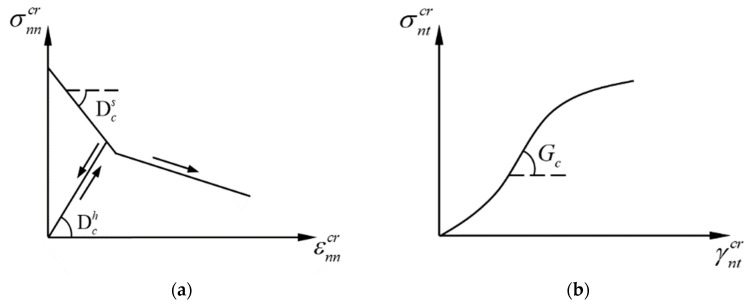
A schematic diagram of the cracking modulus: (**a**) the strain-softening modulus and hysteretic modulus and (**b**) the shear modulus on the crack interface.

Assuming that the stress–strain relationships between the normal and tangential directions of the crack surface are decoupled from each other, the relationship between the stress and cracked strain in the local coordinate system can be written as follows:

(4) $$\left\{\begin{array}{c} \Delta \sigma_{nn}^{cr}\\ \Delta \sigma_{nt}^{cr} \end{array}\right\}=\left[\begin{array}{cc} D_{c} & 0\\ 0 & G_{c} \end{array}\right]\left\{\begin{array}{c} \Delta \varepsilon_{nn}^{cr}\\ \Delta \gamma_{nt}^{cr} \end{array}\right\}$$

where $D_{c}$ is the modulus governing the crack surface opening and closing behavior, and $G_{c}$ is the modulus governing the relative slip between the crack surfaces. Considering the different loading cases, the modulus $D_{c}$ can be further divided into the strain-softening modulus $D_{c}^{s}$, which determines the tensile strain-softening behavior, and the hysteretic modulus $D_{c}^{h}$, which determines the unloading–reloading behavior. The physical meaning of each modulus is illustrated in Figure 4.

Equation (4) can be written in a tensor form as:

(5) $$\Delta s^{cr}=D^{cr}\Delta e^{cr}$$

where $\Delta s^{cr}$ and $\Delta e^{cr}$ are the increment of crack surface stress and strain in the local coordinate system, respectively. $D^{cr}$ is the cracking modulus matrix controlling the complex behavior of the crack surface.

### 2.3. Constitutive Relations of Cracked Concrete in Global Coordinate System

The crack surface stress and crack strain increments in the local coordinate system can be converted into the stress and crack strain increments in global coordinates through coordinate transformation:

(6) $$\left\{\begin{array}{c} \Delta \varepsilon_{xx}^{cr}\\ \Delta \varepsilon_{yy}^{cr}\\ \Delta \gamma_{xy}^{cr} \end{array}\right\}=\left[\begin{array}{cc} \cos^{2}\theta & -\sin\theta \cos\theta \\ \sin^{2}\theta & \sin\theta \cos\theta \\ 2\sin\theta \cos\theta & \cos^{2}\theta -\cos^{2}\theta \end{array}\right]\left\{\begin{array}{c} \Delta \varepsilon_{nn}^{cr}\\ \Delta \gamma_{nt}^{cr} \end{array}\right\}$$

(7) $$\left\{\begin{array}{c} \Delta \sigma_{xx}\\ \Delta \sigma_{yy}\\ \Delta \sigma_{xy} \end{array}\right\}=\left[\begin{array}{cc} \cos^{2}\theta & -\sin\theta \cos\theta \\ \sin^{2}\theta & \sin\theta \cos\theta \\ 2\sin\theta \cos\theta & \cos^{2}\theta -\cos^{2}\theta \end{array}\right]\left\{\begin{array}{c} \Delta \sigma_{nn}^{cr}\\ \Delta \sigma_{nt}^{cr} \end{array}\right\}$$

The formulas (6) and (7) in a tensor form are as follows:

(8) $$\Delta \varepsilon^{cr}=N\Delta e^{cr}$$

(9) $$\Delta \sigma =N\Delta s^{cr}$$

where Δσ and $\Delta \varepsilon^{cr}$ are the stress increment and crack strain increment in the global coordinate system, respectively. N is the coordinate transformation matrix, and θ is the angle between the normal direction of the crack surface and the *x*-axis in the global coordinate system.

The relationship between the stress increment and the total strain increment in the global coordinate system is derived as follows. In the initial undamaged state, the relationship between the stress tensor increment Δσ and the elastic strain tensor increment $\Delta \varepsilon^{e}$ can be written as follows:

(10) $$\Delta \sigma =D^{e}\Delta \varepsilon^{e}$$

where $D^{e}$ is the elastic modulus tensor.

Substituting Equations (3) and (8) into (10):

(11) $$\Delta \sigma =D^{e}[\Delta \varepsilon -N\Delta e^{cr}]$$

Combining Equations (5) and (9):

(12) $$\Delta \sigma =ND^{cr}\Delta e^{cr}$$

Substituting Equation (12) into (11):

(13) $$\Delta e^{cr}={\left[D^{cr}+N^{T}D^{e}N\right]}^{-1}N^{T}D^{e}\Delta \varepsilon$$

Substituting Equation (13) back into Equation (11):

(14) $$\Delta \sigma =[D^{e}-D^{e}N{\left[D^{cr}+N^{T}D^{e}N\right]}^{-1}N^{T}D^{e}]\Delta \varepsilon$$

Equation (14) represents the constitutive equation of the cracked concrete. It can be seen that the constitutive relationship of the cracked concrete is mainly determined by the cracking modulus matrix $D^{cr}$.

### 2.4. Determination of Cracking Modulus

The crack surface strain-softening modulus $D_{c}^{s}$ is determined based on the crack band theory [31,32,33]. The crack band theory assumes that the microcracks are uniformly distributed in a crack band with a width of h. According to the principle of conservation of energy, the relationship between the area $g_{f}$ enclosed by the constitutive curve $\sigma_{nn}^{cr}-\varepsilon_{nn}^{cr}$ and the material fracture energy $G_{f}$ satisfies the following equation:

(15) $$G_{f}=hg_{f}=h\int \sigma_{nn}d\varepsilon_{nn}^{cr}$$

**Figure 5:**
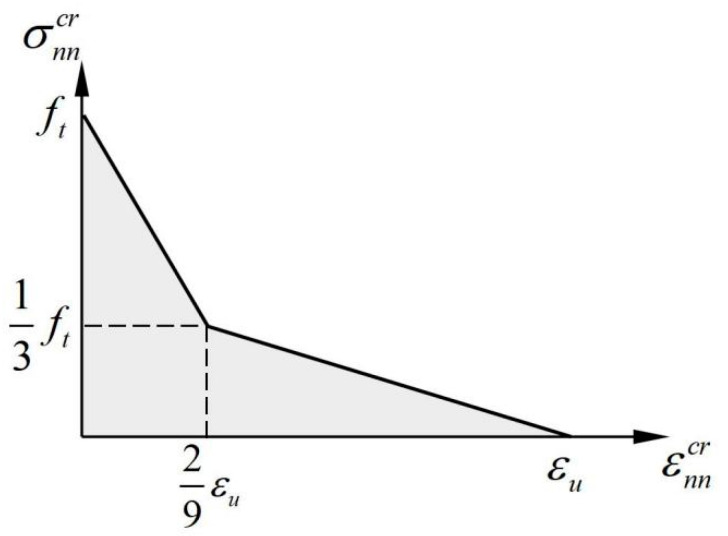
A schematic diagram of the bilinear softening model.

It can be seen that the strain-softening modulus $D_{c}^{s}$ of the crack surface is determined by the parameters $G_{f}$ and h. The parameter h is known as the character length, which is mesh-dependent and affected by the shape, size, type, and integration scheme of the finite element. In the case of a bilinear softening model as shown in Figure 5, the corresponding strain-softening modulus can be calculated as:

(16) $$D_{c}^{s}=\{\begin{array}{c} -\frac{5}{6}\frac{f_{t}^{2}h}{G_{f}}\;\;\;\;\;\;\;\;\;\;\;\;\;\;(0<\varepsilon_{nn}^{cr}<\frac{2}{9}\varepsilon_{u})\\ -\frac{5}{42}\frac{f_{t}^{2}h}{G_{f}}\;\;\;\;\;\;\;\;\;\;\;\;\;\;(\frac{2}{9}\varepsilon_{u}<\varepsilon_{nn}^{cr}<\varepsilon_{u}) \end{array}$$

where $\varepsilon_{u}$ is the ultimate strain. It can be seen that the strain-softening modulus $D_{c}^{s}$ and the ultimate strain $\varepsilon_{u}$ of the softening curve have to be adjusted to the crack band width h.

The hysteretic modulus $D_{c}^{h}$ of the crack surface is determined by the unloading and reloading paths as well as the loading history. In the smeared crack approach, the unloading and reloading paths of concrete under cyclic load can be governed by proposing different hysteretic rules without modifying the constitutive equation. The crack shear modulus $G_{c}$ is usually assumed to be constant, and its value can be determined according to the method in the literature [30].

## 3. Hysteretic Rules Based on Crack Opening–Closing Mechanism

### 3.1. Hysteretic Characteristics of Concrete under Tension–Compression Reversals

**Figure 6:**
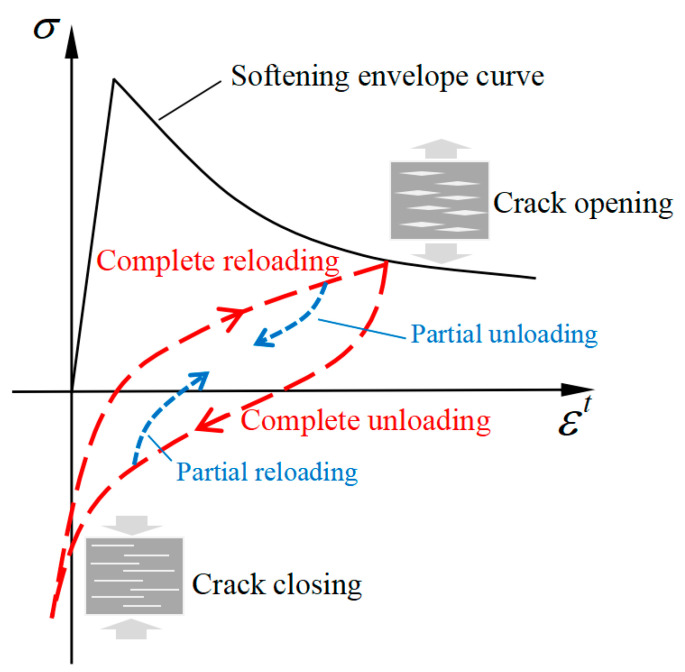
The characteristic hysteretic curve of concrete under tension–compression reversals.

The unloading and reloading behavior of concrete under tension–compression reversals usually presents obvious hysteretic characteristics, accompanied by a large amount of energy dissipation [34,35]. Figure 6 shows the characteristic hysteretic curve of concrete under uniaxial tension–compression reversals, where the horizontal axis represents the tensile strain and the vertical axis represents the tensile stress. In this paper, the loading procedure is defined as the process of the tensile strain increase, and the unloading procedure is defined as that of the tensile strain decrease.

The complete and partial unloading–reloading process under tension–compression reversals is shown as the dotted line in Figure 6. It can be seen that in the process of complete unloading, the stiffness gradually decreases with the reduction in tensile stress, and the inelastic strain is observed when the tensile stress is unloaded to zero. When the stress is reversed, the inelastic strain decreases gradually, while the stiffness increases with the increase in compressive stress. The phenomenon of stiffness recovery under compressive stress is called the “unilateral effect” [36], which is believed to be caused by the complete closure of an open crack under compressive stress [37]. Nouailletas et al. [38] believed that the disappearance of inelastic strain and the recovery of stiffness during crack closure can be partially explained by the friction phenomenon generated by the mismatched discontinuous lips. In addition, the energy dissipated by friction becomes significant for high values of damage and can reach the order of magnitude of the fracture energy.

In the process of complete reloading, the strain increases rapidly and the stiffness decreases gradually. Zhang et al. [39] considered that the hysteretic behavior of concrete under tension–compression reversals is due to the opening and closing process of the crack surfaces.

### 3.2. Proposed Rules for Complete Unloading–Reloading Paths

**Figure 7:**
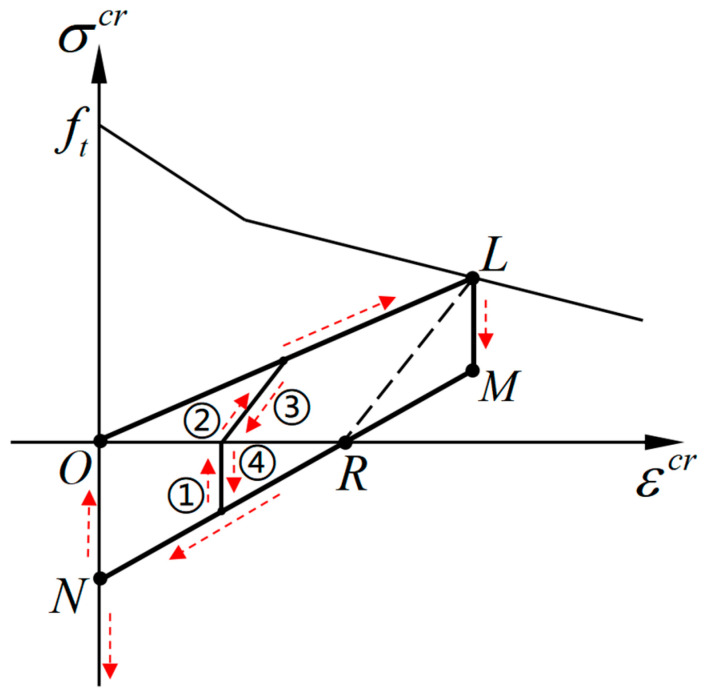
The proposed unloading–reloading paths on the crack interface.

Based on the crack opening–closing mechanism, the hysteretic behavior of concrete under tension–compression reversals can be simulated by constructing the relationship between crack surface stress and crack strain within the framework of smeared crack theory. The hysteretic model is proposed in the local coordinate system to capture the behavior of the crack surface. The complete unloading–reloading paths in the normal direction of the crack surface are depicted in Figure 7, where the horizontal axis represents the crack strain $\varepsilon_{nn}^{cr}$, and the vertical axis represents the normal stress component $\sigma_{nn}^{cr}$ on the crack surface. For simplicity, the subscript “*nn*” of the symbol $\varepsilon_{nn}^{cr}$ and $\sigma_{nn}^{cr}$ indicating the normal direction on the crack surface is omitted. Straight lines are adopted for the unloading and reloading paths. As shown in Figure 7, the complete unloading path is represented by the polyline segments *LMN*, and the complete reloading path is represented by the polyline segments *NOL*.

The complete unloading–reloading paths are determined by three controlling points: point $L(\varepsilon_{un}^{cr},\sigma_{un}^{cr})$ corresponding to the unloading point on the softening envelope curve, point $M(\varepsilon_{un}^{cr},f_{0}^{cl})$ corresponding to the initial closure of the crack surface, and point $N(0,f_{1}^{cl})$ at which the crack surface is completely closed. The coordinate values $f_{0}^{cl}$ and $f_{1}^{cl}$ are called initial closing stress and complete closing stress, respectively, and need to be calibrated through tests. $f_{0}^{cl}$ represents the stress when the crack surface starts to close during the unloading process in tension, and $f_{1}^{cl}$ represents the stress when the crack surface is completely closing under compression.

When all the controlling points have been identified, the modulus $D_{c}^{h}$ of the unloading and reloading paths can be calculated according to the geometric relationship in Figure 7. For the complete unloading path *LMN*:

(17) $$D_{c}^{h}=\{\begin{array}{l} \;\;\;\infty \;\;\;\;\;\;\;\;\;\;\;\;\;\;\;\;(f_{0}^{cl}<\sigma^{cr}\leq \sigma_{un}^{cr})\\ \frac{f_{0}^{cl}-f_{1}^{cl}}{\varepsilon_{un}^{cr}}\;\;\;\;\;\;\;\;\;\;\;\;\;(f_{1}^{cl}<\sigma^{cr}\leq f_{0}^{cl})\\ \;\;\;\infty \;\;\;\;\;\;\;\;\;\;\;\;\;\;\;\;\;\;\;\;(\sigma^{cr}\leq f_{1}^{cl}) \end{array}$$

For the complete reloading path *NOL*:

(18) $$\;D_{c}^{h}=\{\begin{array}{l} \;\infty \;\;\;\;\;\;\;\;\;\;\;\;\;\;(\sigma^{cr}\leq 0)\\ \frac{\sigma_{un}^{cr}}{\varepsilon_{un}^{cr}}\;\;\;\;\;\;\;\;(0<\sigma^{cr}\leq \sigma_{un}^{cr}) \end{array}$$

### 3.3. Proposed Rules for Partial Unloading–Reloading Paths

**Figure 8:**
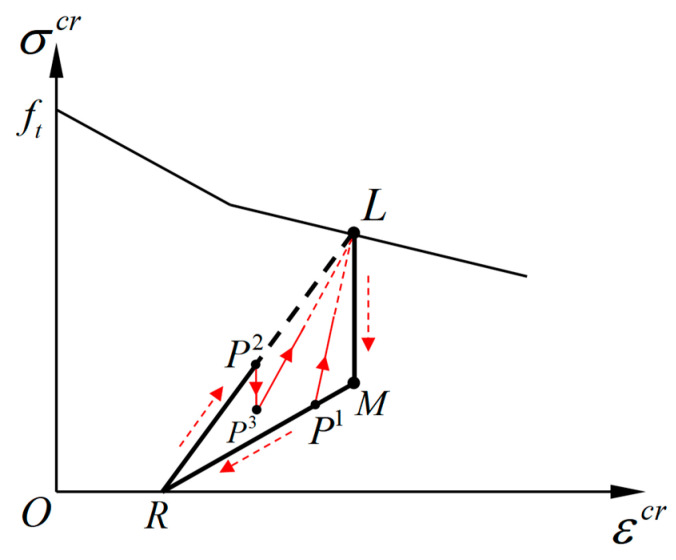
The partial unloading and reloading paths during cyclic tension.

The rules governing the partial unloading–reloading path will be activated when the loading direction reverses during the complete unloading–reloading process. The possible partial unloading–reloading paths under the cyclic tension condition are plotted as shown in Figure 8. Under this condition, the normal stress on the crack surface is always in tension, and the partial unloading–reloading paths always occur in the area enclosed by the polyline segments *LMRL*. The point $R(\varepsilon_{re}^{cr},0)$ is called the residual strain point, whose abscissa values $\varepsilon_{re}^{cr}$ represent the residual crack strain when the normal tensile stress on the crack surface is unloaded to zero.

The strain $\varepsilon_{re}^{cr}$ indicates the deformation due to the incomplete closing of the opening crack surfaces. According to the geometric relationship in Figure 8, the value of $\varepsilon_{re}^{cr}$ is determined by the parameters $f_{0}^{cl}$ and $f_{1}^{cl}$:

(19) $$\varepsilon_{re}^{cr}=-\frac{\varepsilon_{un}^{cr}f_{1}^{cl}}{f_{0}^{cl}-f_{1}^{cl}}$$

Under the condition of the cyclic tension, the rule for the partial reloading path is different from that of the partial unloading path. In the case of unloading, the modulus is specified as equal to 0 as the line segment *P*^2^*P*^3^ shown in Figure 8. The points *P^1^*, *P^2^*, and *P^3^* are called reverse-loading points, which represent the starting points of the partial unloading–reloading paths. The coordinates for the reverse-loading points are uniformly denoted as $P(\varepsilon_{p}^{cr},\sigma_{p}^{cr})$. In the case of reloading, the path starts from the reverse-loading point and is directed to the unloading point, as the lines *P*^1^*L* and *P*^3^*L* shown in Figure 8, and the corresponding modulus is related to the coordinates of the reverse-loading point.

According to the above provisions, the partial reloading and unloading modulus under the cyclic tension can be calculated as follows:

(20) $$D_{c}^{hp}=\{\begin{array}{l} \;\;\;\infty \;\;\;\;\;\;\;\;\;\;\;\;\;\;(\Delta \varepsilon^{cr}\leq 0)\\ \frac{\sigma_{un}^{cr}\;-\;\sigma_{p}^{cr}}{\varepsilon_{un}^{cr}\;-\;\varepsilon_{p}^{cr}}\;\;\;\;\;\;\;\;(\Delta \varepsilon^{cr}>0) \end{array}$$

where the condition $\Delta \varepsilon^{cr}\leq 0$ indicates the unloading process, and the condition $\Delta \varepsilon^{cr}>0$ indicates the reloading process.

Under the condition of tension–compression reversals, all of the possible partial unloading–reloading paths are presented as the dotted line ①, ②, ③, and ④ with an arrow shown in Figure 7. The partial unloading and reloading paths in this condition are specified only dependent on the stress state (tension or compression). According to the geometric relationship, the partial loading and unloading modulus under tension–compression reversals can be calculated as follows:

(21) $$D_{c}^{hp}=\{\begin{array}{l} \;\;\;\infty \;\;\;\;\;\;\;\;\;\;\;(\sigma^{cr}\leq 0)\\ \frac{\sigma_{un}^{cr}}{\varepsilon_{un}^{cr}\;-\;\varepsilon_{re}^{cr}}\;\;\;\;\;\;(\sigma^{cr}>0) \end{array}$$

### 3.4. Determination of the Model Parameters

**Figure 9:**
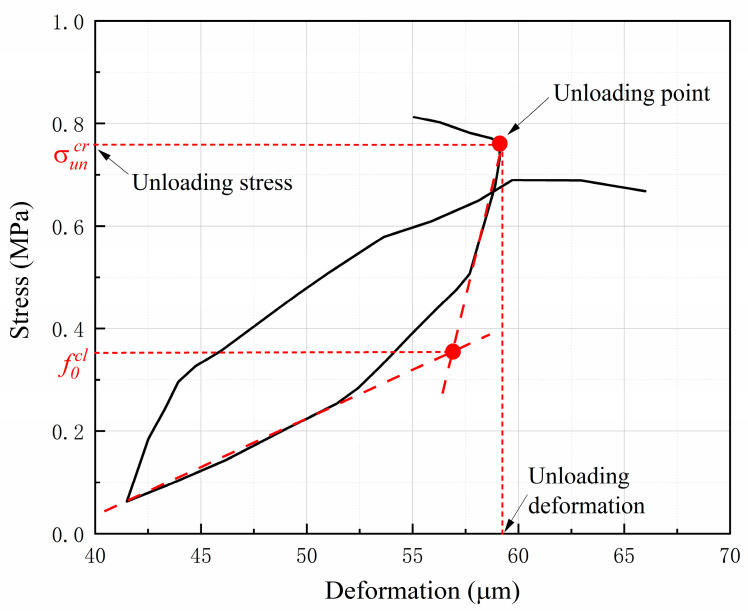
Identification of the initial closing stress from the test curve.

**Figure 10:**
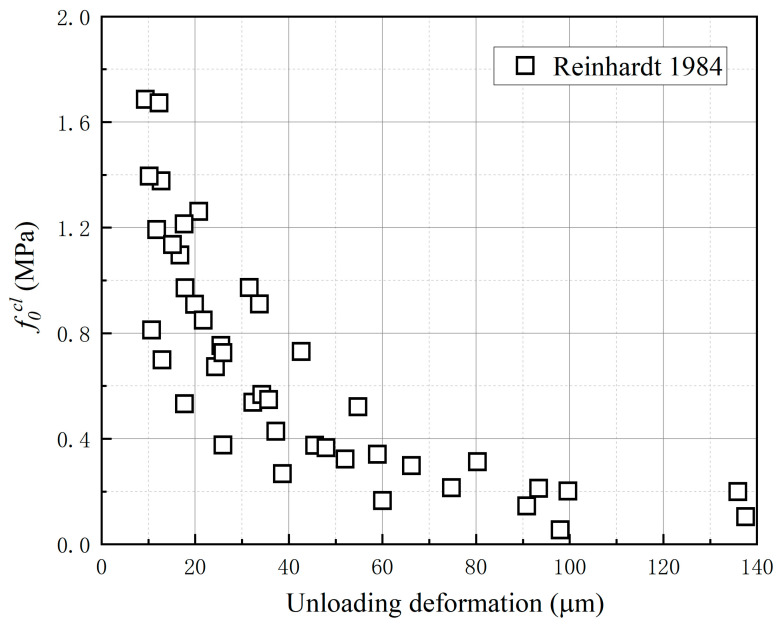
The relationship between the value of $f_{0}^{cl}$ and the corresponding unloading deformations [40].

**Figure 11:**
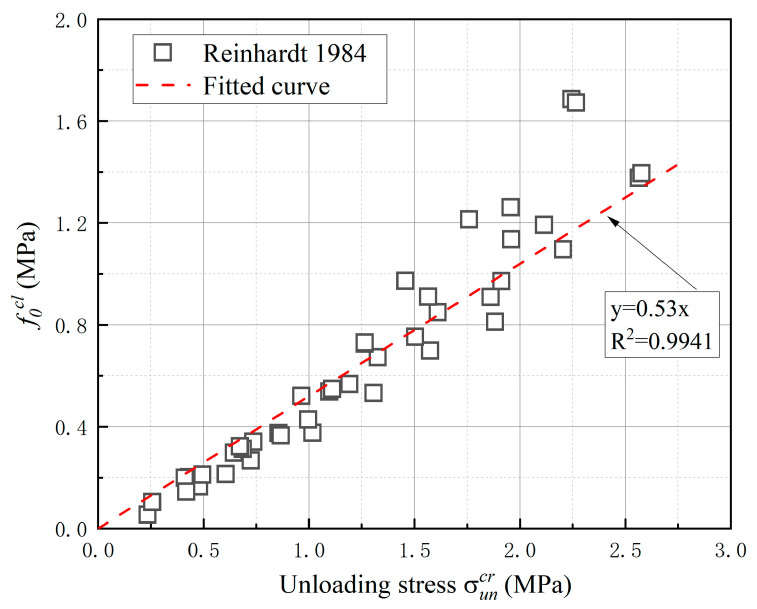
The relationship between the value of $f_{0}^{cl}$ and the corresponding unloading stress [40].

Two model parameters of the proposed unloading–reloading paths are determined in this section. Six stress-deformation curves from the uniaxial cyclic loading tests in the literature [40] were selected to determine the value of the initial closing stress $f_{0}^{cl}$. The value of $f_{0}^{cl}$ corresponding to different unloading points was identified according to the procedure demonstrated in Figure 9. Figure 10 shows the relationship between the value of $f_{0}^{cl}$ and the corresponding unloading deformations. It can be seen that the result appears very scattered and $f_{0}^{cl}$ decreases nonlinearly with the increase in unloading deformation. Figure 11 shows the relationship between the value of $f_{0}^{cl}$ and the corresponding unloading stress $\sigma_{un}^{cr}$. A clear linear feature can be observed in the figure.

Based on the comparison between Figure 10 and Figure 11, it is more reasonable to relate the value of $f_{0}^{cl}$ to the corresponding unloading stress. A fitted linear equation was obtained as shown in Figure 11 and the R-squared of the fitting is equal to 0.9941. The fitted line passes through the origin and the slope is defined as the initial closing coefficient $\alpha =f_{0}^{cl}/\sigma_{un}^{cr}$, which represents the ratio of the initial closing stress to the unloading stress. In the proposed model, the dimensionless parameter α is assumed to be a constant and consequently the value of $f_{0}^{cl}$ is determined by the corresponding unloading stress $\sigma_{un}^{cr}$.

For the determination of the complete closing stress $f_{1}^{cl}$, there is no unified conclusion at present. In the literature [39], the value of $f_{1}^{cl}$ was set as one-third of tensile strength based on the experimental results. In the literature [40], the value of $f_{1}^{cl}$ was considered related to the degree of compressive damage in concrete.

### 3.5. Numerical Implementation of the Proposed Model

**Figure 12:**
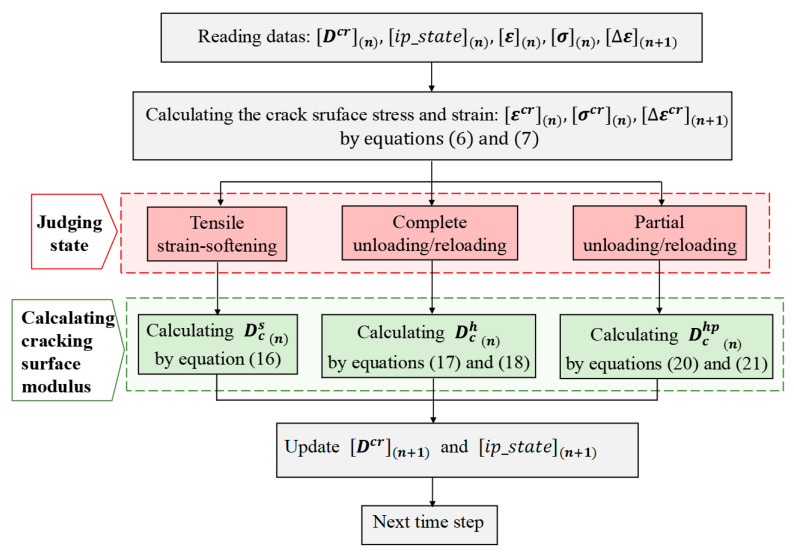
A flow chart of the computational implementation of the proposed model.

According to the hysteretic rules proposed in this paper, the unloading and reloading paths of the model can be implemented by modifying the cracking modulus within the framework of smeared crack theory. The numerical algorithm of the proposed model is implemented in a special finite element program using Matlab. The flow chart for updating the crack surface modulus is shown in Figure 12.

## 4. Simulation Results and Discussion

### 4.1. Cyclic Tests of Concrete

**Figure 13:**
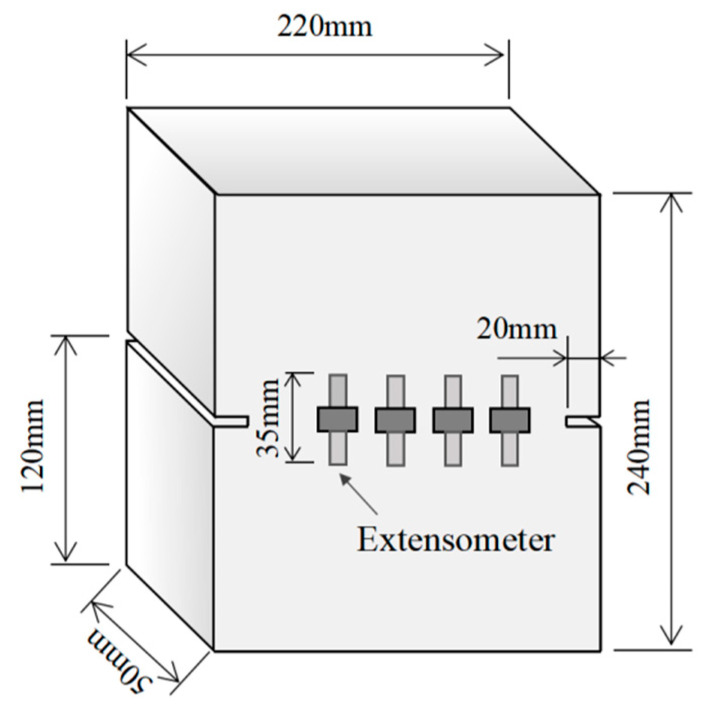
The schematic diagram of the concrete specimen.

The concrete cyclic tests conducted by Reinhardt [40] were chosen in this study to validate the proposed model. The average tensile strength of concrete was 3.2 MPa, the average elastic modulus was 39.3 GPa and the fracture energy was 132 N/m. The specimens for cyclic tension and tension–compression tests were concrete prisms with dimensions of 240 mm × 220 mm × 50 mm. Two grooves with a depth of 20 mm were precut on both sides of the specimen along the thickness direction to control the initial position of cracking. The schematic diagram of the specimen is shown in Figure 13.

The cyclic loading procedure was displacement-controlled, and three cyclic loading programs T1, T2, and T3 with different stress ranges were adopted. The lowest stress in cyclic loading program T1 was the tensile stress with an amount equal to 5% of the tensile strength. The lowest stress in loading program T2 was the compressive stress with an amount equal to 15% of the tensile strength. The lowest stress in loading program T3 was the compressive stress with the amount equal to the tensile strength. Four extensometers with a gauge length of 35 mm were installed on the front and back of the specimen to measure the deformation during the cracking process. The arrangement of the measuring device is shown in Figure 13. Based on the measured test data, the complete stress-deformation curves during cyclic loading were obtained.

**Figure 14:**
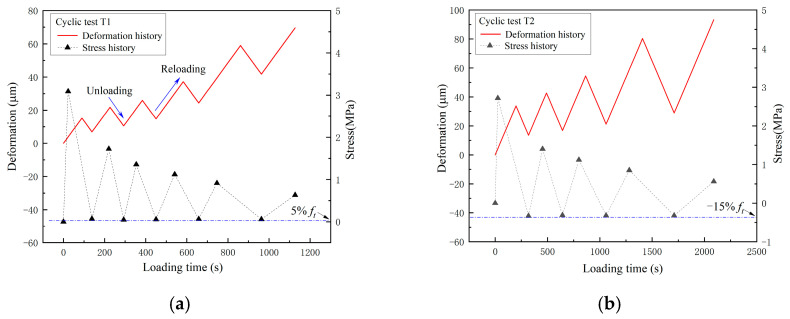

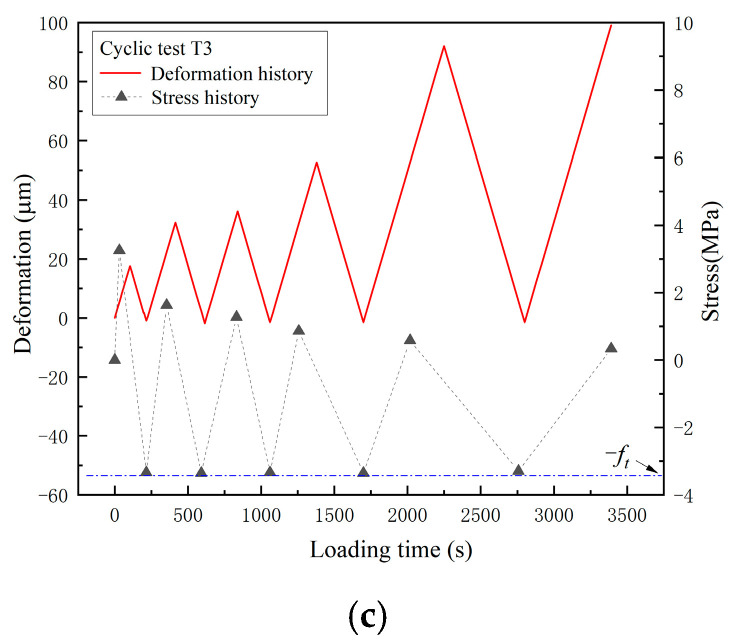
The cyclic loading history of programs (**a**) T1, (**b**) T2, and (**c**) T3.

Figure 14 shows the history of deformation measured by the extensometers in the cyclic loading programs T1, T2, and T3. The decrease in deformation is defined as unloading and the increase in deformation is defined as loading. The extreme value points of the stress history are also plotted in the figure. It can be seen that under the three loading programs, the maximum value of stress decreases with cyclic loading, while the minimum value of stress is stable at the design value.

### 4.2. Numerical Model and Parameters

**Figure 15:**
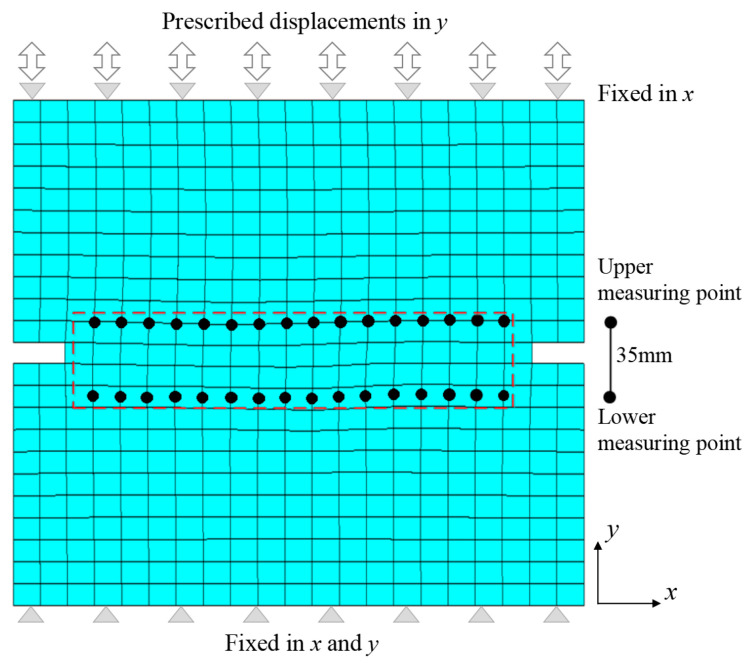
The finite element mesh of the notched specimen.

According to the constraints and loading conditions, the specimen is always in a plane stress state during the cyclic loads, so the two-dimensional finite element model is adopted, as shown in Figure 15. The finite element mesh of the notched specimen is shown in the figure. The meshes consist of 528 nodes and 479 four-node linear elements, numerically integrated by means of the four-point Gaussian scheme. There is no constraint on both sides of the specimen. The bottom surface of the specimen is fixed, and the top surface is fixed in the *x* direction and moves in the *y* direction according to the prescribed displacement history.

The following material properties are adopted for concrete: Young’s modulus *E_0_* = 39.3 GPa, Poisson’s ratio *v* = 0.2, tensile strength *f_t_* =3.2 MPa, and fracture energy *G_f_* = 132 N/m. The hysteretic model is introduced into the concrete constitutive relationship. Based on the experimental data of Reinhardt’s test, the model parameters α and $f_{1}^{cl}$ are set to be 0.53 and *f_t_*/3, respectively.

**Figure 16:**
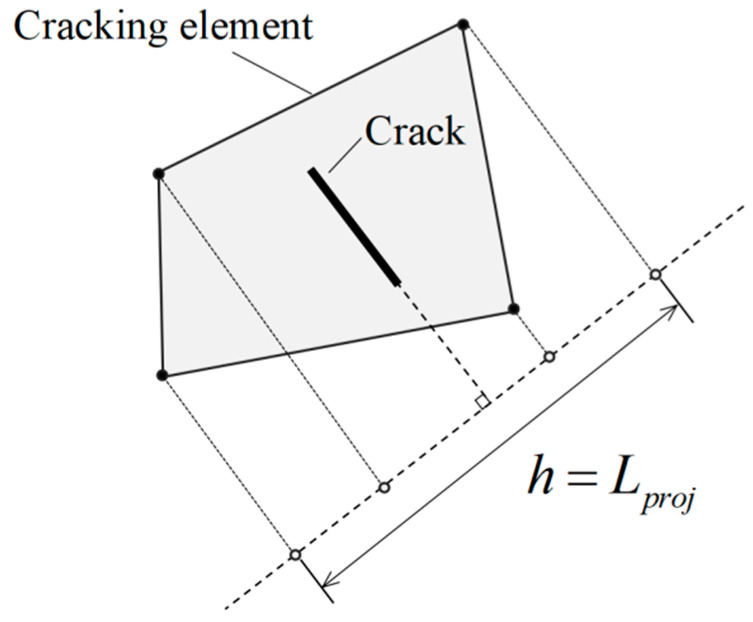
Calculation method of crack band width.

The crack band theory is used to guarantee the proper energy dissipation during the fracturing process. A bilinear softening relationship is adopted in this paper. In order to reduce the dependency of results on finite element mesh sizes, the width of crack band *h* is calculated for each finite element as the element length projected into the normal crack direction [41], as depicted in Figure 16.

### 4.3. Simulation Results of Direct Tension Test

#### 4.3.1. The Stress-Deformation Curve

**Figure 17:**
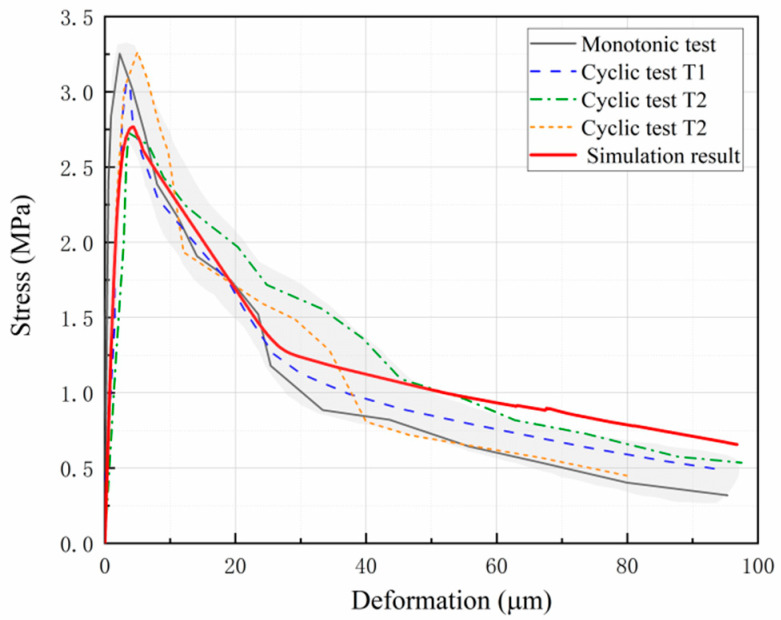
Comparison of the stress-deformation softening curves.

The test and simulation results of the tensile stress-deformation softening curve of concrete are shown in Figure 17. The test results include data from monotonic tensile tests and the tensile-softening envelopes obtained from cyclic loading tests. The deformation in the test results was obtained by averaging the elongation measured by all the extensometers mounted on the surface of the specimens. Accordingly, the deformation of the simulation results was obtained by calculating the average longitudinal relative displacement of the area where the extensometers were distributed. The simulated stress-deformation curve reproduces the nonlinear characteristics during the stress rise phase and the softening characteristics after the peak stress. A good agreement is observed between the simulated curve and the test curves.

#### 4.3.2. Evolution of Deformation Distribution

**Figure 18:**
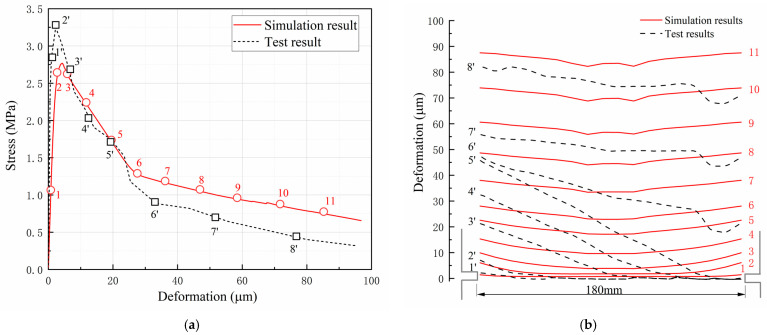
Comparison of deformation distribution curves: (**a**) the characteristic points and (**b**) the tensile stress distribution curves.

Figure 18 shows the test and simulation results of the evolution of the deformation distribution in the monotonic tensile test. The deformation distribution curves along the cross section at the groove in Figure 18b correspond to the characteristic points marked on the tensile stress-deformation curves in Figure 18a. Eight test curves and eleven simulation curves were plotted to compare the evolution characteristics of the deformation distribution during the tensile test.

In the simulation results, it can be seen that the deformation distribution curves are always symmetrical. In the elastic stage of initial loading (point 1), the deformation is evenly distributed along the section of the groove. As the tensile stress increases, the deformation near the groove increases rapidly, and the deformation distribution curve evolves into a “U” shape before the stress reaches the peak (point 2). The large deformation near the groove indicates the initiation of the crack. When the stress-deformation curve enters the softening stage, the deformation increases rapidly and gradually evolves to an approximatively uniform distribution (points 3–11). The evolution characteristics of the simulated deformation distribution curves show that the crack originated from the groove at both ends and extended to the middle simultaneously.

In the test results, an asymmetric distribution of deformation is observed, which differs significantly from the simulated results. From initial loading to the first stage of softening, the deformation near the groove on one side increases rapidly, but the deformation near the groove on the other side is almost zero (points 1′ to 5′). It is not until the stress deformation curve enters the second stage of softening that the deformation gradually evolves to an approximatively uniform distribution (points 6′ to 8′). The evolution of the test deformation distribution curves shows that the crack initiated from one side of the groove and gradually expanded to the other side. This result may be due to the unevenness of the tensile force applied to the specimen during the test.

#### 4.3.3. Simulation Results of Stress and Strain Distribution

**Figure 19:**
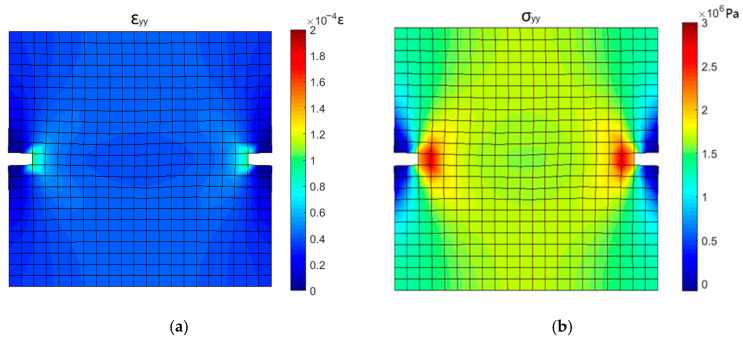
Stress and strain contours corresponding to point 1: (**a**) *y*-directional strain and (**b**) *y*-directional stress.

**Figure 20:**
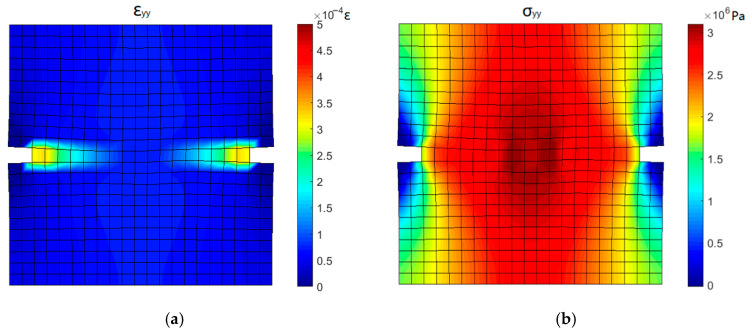
Stress and strain contours corresponding to point 3: (**a**) *y*-directional strain and (**b**) *y*-directional stress.

**Figure 21:**
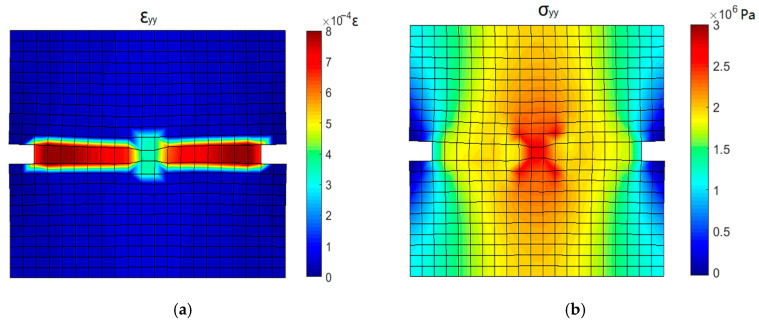
Stress and strain contours corresponding to point 5: (**a**) *y*-directional strain and (**b**) *y*-directional stress.

The simulation results of the contour plots of stress and strain in the *y*-direction corresponding to state points 1, 3, and 5 in Figure 18a are shown in Figure 19, Figure 20 and Figure 21.

Figure 19 shows the stress and strain contours corresponding to the linear elastic stage of the stress-deformation curve (point 1). A significant concentration of stress and strain is observed near the groove. At the top and bottom ends of the specimen, the stress in the middle is obviously higher than that on both sides due to the uneven resistance caused by the groove.

Figure 20 shows the stress and strain contours when the stress crosses the peak and begins to decrease (point 3). The strain at the grooves increases rapidly and the high strain zone is extending from the groove to the middle of the specimen. The stress near the groove decreases due to the occurrence of a crack and the position of the maximum stress shifts to the middle of the specimen. High stress is spread throughout the specimen, and the load carried by the specimen reaches the maximum value.

Figure 21 shows the stress and strain contours corresponding to the softening stage (point 5). The strain at the groove section increases rapidly and a distinct “crack band” is observed almost across the groove section. As the crack extends toward the middle of the specimen, the stress near the groove decreases, and an “X”-shaped high-stress zone is observed in the middle of the specimen.

### 4.4. Simulation Results of Cyclic Tension–Compression Test

#### 4.4.1. The Stress-Deformation Curves

**Figure 22:**
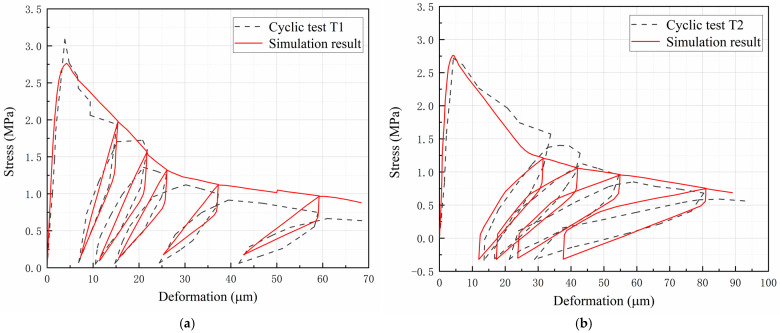

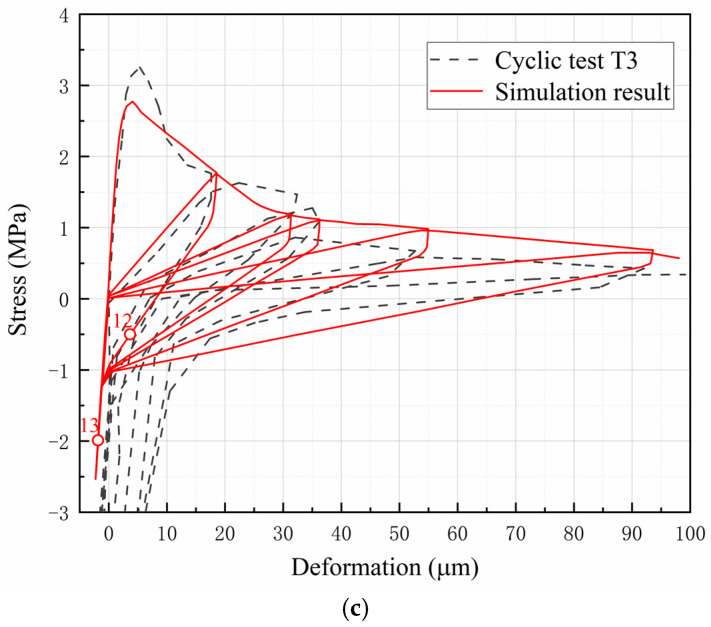
Comparison between test and simulation results of (**a**) cyclic test T1, (**b**) cyclic test T2, and (**c**) cyclic test T3.

Figure 22 shows comparisons between the test and simulation results of cyclic tests. Figure 22a shows the test and simulation results of the stress-deformation curves under cyclic loading condition T1. It can be seen that the unloading and reloading curves do not coincide, and they enclose a spindle-shaped closed loop, which is commonly known as the “hysteresis loop”. The presence of the hysteresis loop indicates that there is additional energy dissipated during the unloading–reloading process. According to the mechanism of the hysteretic behavior of concrete, the dissipated energy is considered to be related to crack propagation and friction behavior of crack surfaces. The simulation results of the hysteresis loop during the first softening stage agree very well with the test results. During the second softening stage, deviations between simulated hysteresis loops and test results are observed, which are caused by the offset of the simulated softening envelope. Although the unloading and reloading paths in the model are specified as linear segments, the simulated stress-deformation curve exhibits nonlinear characteristics similar to the test results.

Figure 22b shows the test and simulation results of the stress-deformation curves under cyclic loading condition T2. In this cyclic test, the loading direction changes during the unloading and reloading process, and the stress state alternates between tension and compression. The hysteresis loops under the loading condition T2 are more significant compared to the loading condition T1, indicating that more energy is dissipated during the unloading and reloading process. As can be seen, the four hysteresis loops predicted by the model are in good agreement with the test results.

Figure 22c shows the test and simulation results of the stress-deformation curves under cyclic loading condition T3. In this condition, the compression stiffness recovers to the initial value when the compressive stress reaches the crack-closing stress. The stress-deformation curve presents complete unloading and reloading paths with a large amount of energy dissipated. It can be seen that the simulation results of the hysteresis loop are greatly influenced by the simulation accuracy of the stress-deformation envelope. Because the unrecoverable tensile deformation is not considered in the model, the simulated curve in the compression zone deviates from the test results.

#### 4.4.2. Stress and Strain Contours of Crack-Closure Process

**Figure 23:**
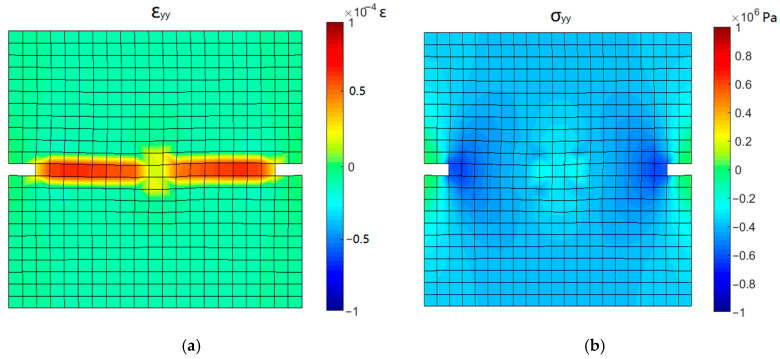
Stress and strain contours corresponding to point 12: (**a**) *y*-directional strain and (**b**) *y*-directional stress.

**Figure 24:**
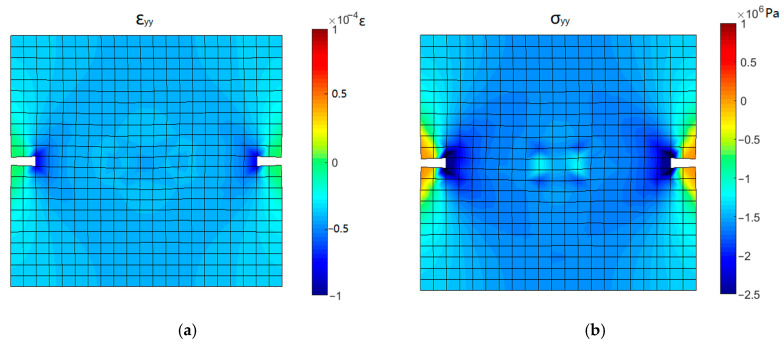
Stress and strain contours corresponding to point 13: (**a**) *y*-directional strain and (**b**) *y*-directional stress.

In cyclic test T3, with the increase in compressive stress, the open crack surfaces gradually closed. The stress and strain contours corresponding to the state points 12 and 13 during the process of crack closure in Figure 22c are shown in Figure 23 and Figure 24.

During the initial stage of reversed compression (point 12), the specimen is in compression and the concentration of compressive stress is observed near the groove. Since the crack is not completely closed, the cross-section at the groove still retains a large tensile strain, while the tensile strain of other parts has been released. When the compressive stress increases to 2 MPa (point 13), the compressive strain is observed in the cross-section at the groove, indicating the complete closure of the crack surface. The distribution of compressive strain in the specimen is the same as that of compressive stress, which means that the sample entered the state of elastic compression.

#### 4.4.3. Evolution of Stiffness and Dissipated Energy

**Figure 25:**
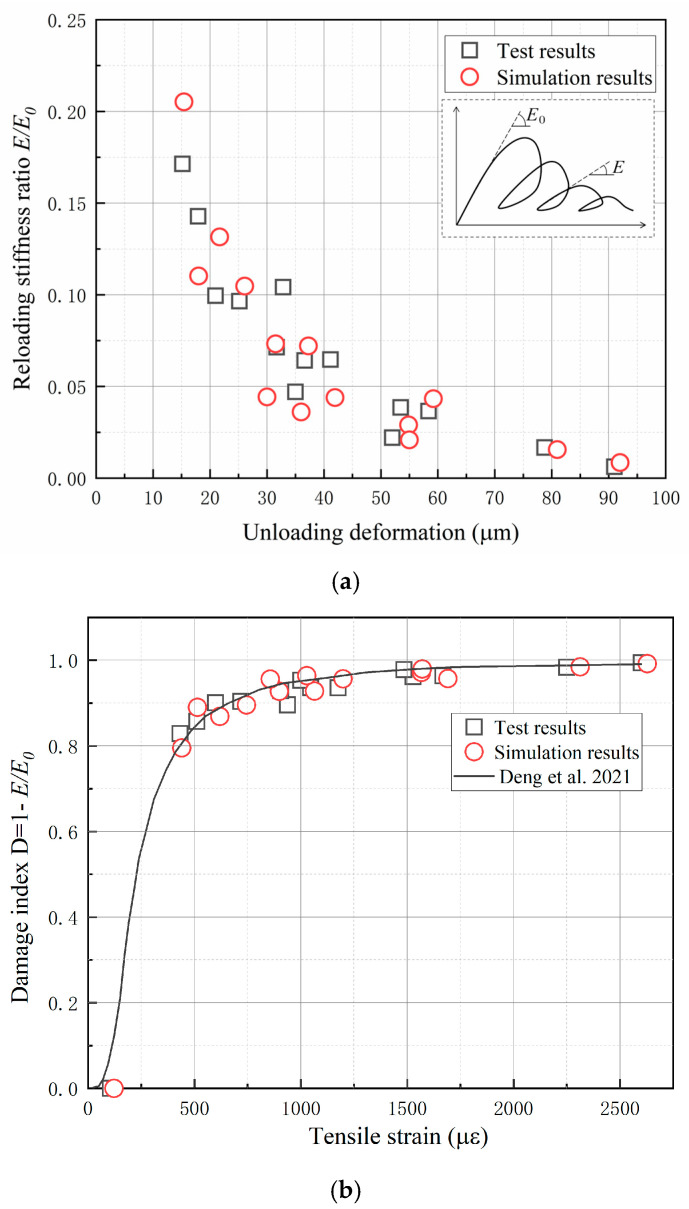
The test and simulation results of (**a**) reloading stiffness ratio and (**b**) damage index [42].

The test and simulated results of the reloading stiffness ratio are compared in Figure 25a. The reloading stiffness ratio is defined as the ratio of the reloading stiffness *E* to the initial loading stiffness *E_0_* in the cyclic load test, as illustrated in Figure 25a. The reloading stiffness ratios in the cyclic load tests T1, T2, and T3 were calculated and summarized in Figure 25a. It can be seen that the reloading stiffness ratio decreases rapidly with the increase in deformation. When the deformation reaches 20 μm, the reloading stiffness declines to 15% of the initial loading stiffness. When the deformation reaches 50 μm, the reloading stiffness declines to less than 5% of the initial loading stiffness. The simulated reloading stiffness ratio presents good agreement with the test results.

According to the continuum damage mechanics theory, the damage of material can be characterized by reloading stiffness ratio. Figure 25b shows the test and simulation results of the damage corresponding to different unloading strains in the cyclic tests. The unloading tensile strain in the figure was obtained by dividing the unloading deformation by the extensometer length of 35 mm. In addition, the damage at peak strain is assumed to be zero. As can be seen from the figure, the damage increases rapidly after entering the softening stage, reaching 0.8 at 500 με. After that, the growth rate gradually decreases and gradually tends to 1. As a comparison, the damage evolution curve based on the analytic envelope expression in the literature [42] was also drawn in the figure. It can be seen that the damage simulation results at different unloading strains are very consistent with the test data and the damage evolution curve. This indicates that the hysteretic model has a good ability to predict the damage evolution feature under cyclic tension.

Further comparison is performed to validate the proposed model. The predicted dissipated energy during each cycle of the tests T1, T2, and T3 is compared with the test results in Table 1. The magnitude of the dissipated energy of each cycle was obtained by calculating the area of the corresponding hysteresis loop. For the cyclic tests T2 and T3, the hysteretic dissipated energy increases with the increase in the cyclic ordinal number (i.e., unloading deformation). Good agreement is observed between the dissipated energy results of the test and simulation. In the case of cyclic tests T1 and T3, the relative errors of the total cumulative dissipated energy are only 2.37% and 7.05%, respectively.

## 5. Conclusions

Based on the smeared crack theory, this paper presents a hysteretic model to describe the nonlinear constitutive relationship of concrete under cyclic tension and tension–compression reversals. The numerical predictions of the proposed model are compared with several test results, and the following conclusions are obtained.

(1) By modifying the cracking modulus, the opening–closing behavior of the crack surface that produces the hysteretic phenomenon of concrete under cyclic tension–compression is directly modeled in the framework of the smeared crack theory.
(2) The proposed model is able to reproduce the hysteretic curves of concrete under complex tensile cyclic load conditions, as well as the degradation of reloading stiffness, energy dissipation, and stiffness recovery due to the crack closure.
(3) The model can simulate the initiation and propagation of concrete cracks under uniaxial cyclic tensile load, as well as the opening and closing behavior of the crack surface during the unloading–reloading process.
(4) The model adopts linear unloading–reloading paths and has only two parameters, which make the model easy to use and suitable for introduction into finite element programs.
(5) The model was verified by comparing the results with several cyclic tests. In addition, the proposed model can be applied to the nonlinear analysis of real concrete structures.

## Figures and Tables

**Table 1:** Comparisons of test and simulated dissipated energy results.

Cycle Number	Cyclic Test T1			Cyclic Test T2			Cyclic Test T3		
	Test (10^−3^ N/mm)	Simulation (10^−3^ N/mm)	Error (%)	Test (10^−3^ N/mm)	Simulation (10^−3^ N/mm)	Error (%)	Test (10^−3^ N/mm)	Simulation (10^−3^ N/mm)	Error (%)
1	2.45	2.44	−0.16	7.37	9.37	−21.4	15.68	15.23	−2.94
2	3.31	2.96	−11.6	7.74	11.32	−31.6	25.11	22.54	−11.4
3	2.12	2.45	13.3	8.75	13.43	−34.8	27.1	26.27	−3.18
4	2.28	2.24	−1.66	12.39	17.33	−28.5	32.77	40.5	19.1
5	2.87	2.62	−9.27	-	-	-	51.05	58.69	13.0
Total	13.03	12.72	−2.37	36.24	51.44	−29.5	151.71	163.22	7.05

## Data Availability

Some or all data, models, or code that support the findings of this study are available from the corresponding author upon reasonable request.
